# Deploying a military emergency medical team in flood response: operational lessons from Orkadiéré, Senegal

**DOI:** 10.1016/j.afjem.2026.101023

**Published:** 2026-11-07

**Authors:** Abdoulaye Sow, Arona Solar Niane, Modou Niane, Mohamed Lamine Coly

**Affiliations:** a Médecin-Commandant, Service de Santé des Armées & Hôpital Principal de Dakar, Dakar, Sénégal; b Médecin-Capitaine, Service de Santé des Armées & Hôpital Principal de Dakar, Dakar, Sénégal; c Médecin-Lieutenant, Service de Santé des Armées & Hôpital de Fann, Dakar, Sénégal; d Lieutenant, Corps Technique et Administratif, Service de Santé des Armées, Sénégal

**Keywords:** Disaster response, Flood, Military medicine, Emergency medical team, Humanitarian response, Senegal

## Abstract

In October 2024, exceptional flooding along the Senegal River, exacerbated by unusual rainfall and dam releases from the Manantali and Diama infrastructures, directly affected approximatively 55,600 people across the Matam, Saint-Louis and Tambacounda regions and severely disrupted healthcare access in dozens of inundated communities. The Senegalese Armed Forces deployed a World Health Organization Type 1 Emergency Medical Team (EMT) to Orkadiéré, a strategic locality in the Matam region selected through a joint pre-deployment assessment with local civilian health and administrative authorities. Over 16 days (16–31 October 2024), a ten-member team comprising three physicians, a senior nurse, two nurses, two nursing assistants, an administrative officer and a driver—supported by dedicated military logistics and security units—delivered 360 documented medical consultations, conducted field missions to riverine communities accessible only by pirogue, and maintained active epidemiological surveillance for epidemic-prone diseases. This commentary reflects on four operational lessons derived through formal after-action reporting within the military chain of command: joint pre-deployment assessment for site selection; multidisciplinary team composition; separation of logistical from clinical functions; and epidemiological surveillance from mission day one. It also examines the civil-military coordination model and implications for institutionalising military humanitarian medical capacity within the Senegalese Armed Forces.

## African relevance

• Flood response lessons are relevant to emergency care across Africa.
• Military EMTs can extend care to isolated riverine communities.
• Civil-military coordination can strengthen disaster health response.
• Early surveillance supports outbreak detection after flooding.

## Introduction

Natural disasters are increasing in frequency and severity across sub-Saharan Africa, driven by climate variability and structural vulnerability in flood-prone regions. Senegal experiences recurrent seasonal flooding, with northern territories traversed by the Senegal River particularly exposed. In October 2024, the convergence of exceptional rainfall and water releases from the Manantali and Diama dams produced flooding of unprecedented scale: more than 44 sites in the Matam region and 51 villages in Saint-Louis were inundated, directly affecting approximately 55,600 people [1,2]. Healthcare access was severely disrupted, with multiple communities reachable only by pirogue.

Military medical teams represent a critical surge capacity in such settings, combining rapid deployment capability, logistical self-sufficiency and operational discipline under austere conditions [3]. The World Health Organization Emergency Medical Team (EMT) classification framework provides a common reference for planning, coordination and accountability across humanitarian medical responses [4]. Despite this, published accounts of military EMT operations in West African flood settings remain scarce, limiting peer learning and evidence-based planning [5].

This commentary describes the deployment of a Senegalese Armed Forces Level (Type) 1 EMT to Orkadiéré during the October 2024 floods. We present the operational context, activities and lessons learned, with the aim of contributing a structured operational narrative that may inform planning for similar deployments across flood-prone African contexts. This is not an original research report: it is operational testimony from practitioners, offered with transparency about its methodological limitations.

### The deployment: context and level 1 operations

EMTs are classified by the World Health Organization according to clinical capability and self-sufficiency. Type 1 teams provide ambulatory and emergency primary care; Type 2 teams add inpatient surgical capacity; and Type 3 teams function as referral hospitals [4]. The Orkadiéré deployment constituted a Type (Level) 1 EMT, designed for outpatient consultations, triage, basic emergency treatment and stabilisation, without inpatient beds or operative capability. This scope was appropriate to the anticipated needs of the affected population: widespread primary-care disruption, elevated communicable-disease burden and community isolation, rather than mass-casualty trauma.

Site selection involved a rapid joint assessment with the district medical officer and the regional governor. Three localities—Thiagnaff, Kanel and Ogo—were initially considered. Orkadiéré was selected for its central geographic position relative to the most severely affected zones and the pre-existing presence of a fire brigade diving team. The deployment area is shown in Fig. 1. The diving team was integrated into the operational plan primarily to provide aquatic access to riverine communities. In practice, its principal contribution was logistical—facilitating pirogue-based access to isolated villages—rather than technical rescue. No aquatic rescue operations were conducted during the deployment.

**Fig. 1:**
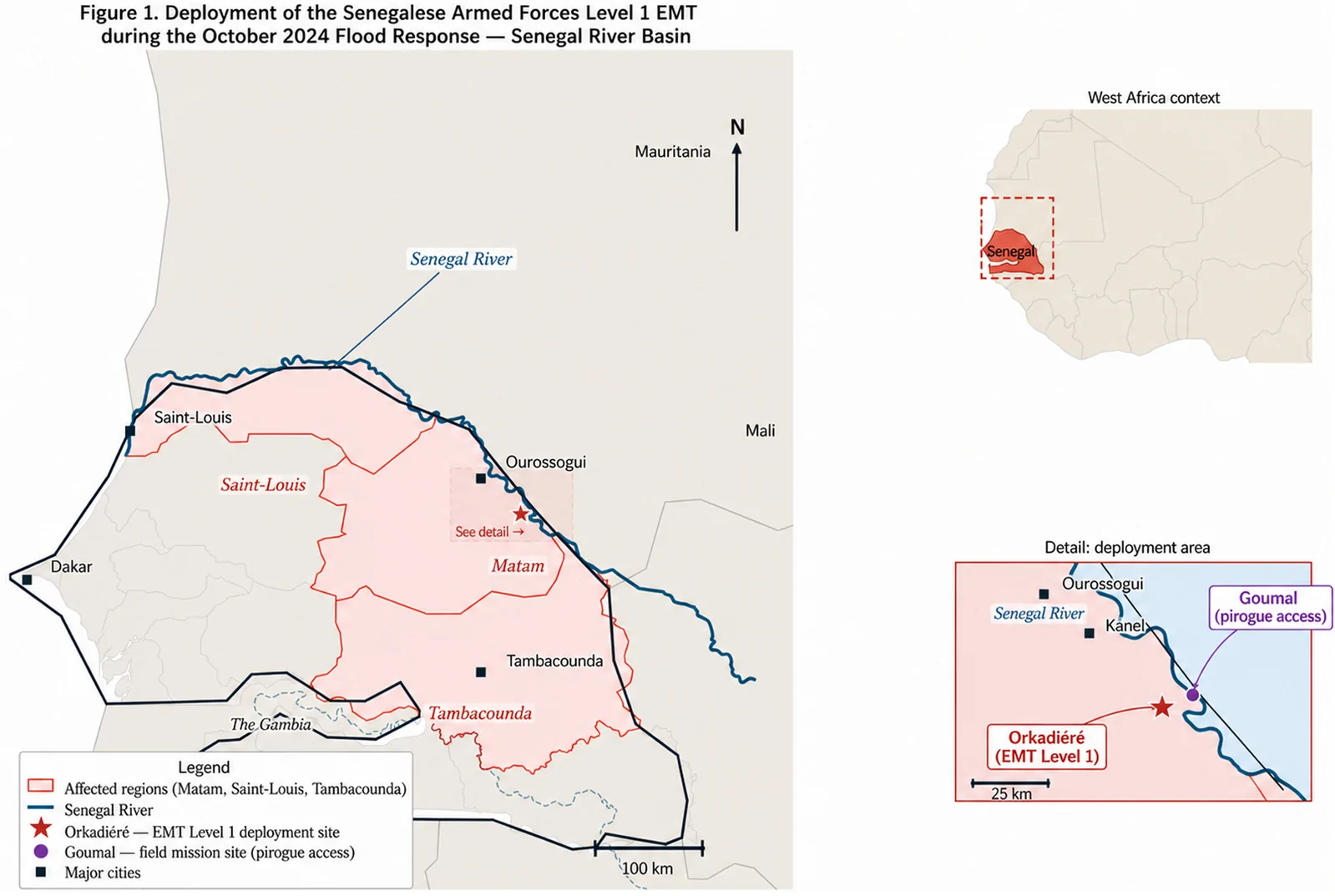
Location of Orkadiéré, Matam Region, Senegal, and the Senegal River basin.

The Level 1 post was operational within 72 h of mobilisation, a pace enabled by advance logistics coordination and pre-positioned materiel. Integration with the district health system ensured continuity with existing care infrastructure rather than parallel operation, and sustained the community trust that is foundational to effective care-seeking in resource-limited settings.

## Team composition and civil-military coordination

The Matam EMT comprised ten members. Clinical personnel included an anaesthesiologist-resuscitationist serving as team leader, an emergency physician and a general practitioner. Nursing staff consisted of one senior nurse (infirmier-major), two nurses and two nursing assistants. Non-clinical support was provided by an administrative and technical officer (OCTASSA) and a driver, both integral to the team’s operational functioning.

Logistical and security support was provided by four dedicated military units: the 32nd Infantry Battalion of Ourossogui, the Directorate of Materiel (DIRMAT), the Transport Battalion (Bataillon du Train) and the Directorate of Engineering (DIRGEN). All four units were elements of the Senegalese Armed Forces, deployed under the authority of the État-Major Général des Armées (EMGA). The deployment operated through the chain of command of the Direction du Service de Santé des Armées (DSSA), with mission coordination on the ground facilitated by the Commandant de Zone Militaire No. 2 (ComZone 2), who conducted a joint field visit to the Level 1 site and to the isolated village of Goumal on 22 October 2024. Clinical operations were managed autonomously by the medical team commander (Chef EMU), with progress reported to the DSSA through formal Comptes Rendus.

Coordination with civilian authorities was sustained throughout the deployment. Site selection was co-determined with the district medical officer and the regional governor; ongoing liaison was maintained with the sous-préfet of Orkadiéré; and the Minister of Hydraulics and Sanitation, accompanied by the Governor of Matam, visited the Level 1 site. This model—military logistical and security capability deployed under civilian health-authority oversight, with a clear command interface at zone level—is a recognised configuration for effective civil-military health response [6]. By separating the military’s operational role (logistics, security and access) from the civilian health system’s normative role (care standards, reporting and community accountability), the deployment avoided coordination tensions that commonly weaken such collaborations in humanitarian settings.

## Medical activities: scope, data and surveillance

The Level 1 post delivered 360 documented consultations—the official count recorded in the formal after-action Compte Rendu submitted to the DSSA on 3 November 2024—for inhabitants of Orkadiéré and surrounding villages, including Gouriky-Golliabé, Verma and Wendou Bosséabé. The principal pathologies addressed were malaria, acute respiratory infections, wound care and waterborne disease-related presentations, a pattern consistent with the post-flood epidemiological profile reported in the humanitarian literature [7].

Consultation data were recorded in operational field registers and consolidated in formal Comptes Rendus submitted through the military chain of command to the DSSA. Prospective disaggregation by category—new flood-related presentations versus routine care-seeking, traumatic versus non-traumatic, and paediatric versus adult—was not systematically performed. Consequently, the disease burden attributable specifically to the disaster cannot be isolated from background community health need. This is a substantive methodological limitation: future deployments should integrate standardised data-collection instruments, and the World Health Organization Health Resources Availability Mapping System, from mission day one to generate actionable epidemiological evidence.

Active surveillance for epidemic-prone diseases was maintained throughout the deployment, with daily clinical monitoring for signs of cholera, acute diarrhoeal disease and dengue. Findings were reported through the formal Compte Rendu mechanism to the DSSA. No outbreak was detected during the deployment period. The Compte Rendu for the Goumal mission (23 October 2024) documented that the village health post was functioning, with consultation volumes stable despite displacement and no flood-related deaths recorded at the time of assessment.

A dedicated field mission reached Goumal on 22 October 2024, traversing the villages of Gangel Souley, Padalal and Barkewi before arriving at the fully water-surrounded village, accessible only by pirogue. Approximately 60 displaced families were sheltered near the local health post. The team conducted consultations, reinforced the health post’s medication stock and documented a community protection concern: schoolchildren could not safely access the secondary school because of flooding. The Commandant de Zone Militaire No. 2, who joined the mission, committed to supplying life jackets for students and escalating the access problem to higher authorities. This integration of clinical care with community protection advocacy illustrates a distinctive feature of military humanitarian deployments: the command authority to act beyond the purely medical mandate.

## Lessons learned: process and substance

### Deriving the lessons

The lessons presented in this commentary were derived through the formal after-action reporting process of the Senegalese Armed Forces. Two structured Comptes Rendus were submitted by the mission commander to the Directeur du Service de Santé des Armées during and immediately after the deployment: a field report on the Goumal mission (23 October 2024) and a comprehensive medical activity report (3 November 2024). These reports, produced within the military chain of command, constitute the primary documented source for the lessons presented here. They represent the operational judgment of the deploying medical team leadership rather than the output of an independent external evaluation—a limitation that future deployments should address by complementing internal reporting with structured multi-stakeholder debriefs.

### Lesson 1—Joint pre-deployment assessment determines mission success

The decision to deploy to Orkadiéré rather than any of the three initially considered alternatives was shaped by a joint field assessment co-conducted with local health and civilian authorities. This process served a dual function: it produced technically sound site-selection criteria and established relational legitimacy before the team arrived. In resource-constrained settings where trust directly conditions care-seeking behaviour, community legitimacy is foundational to mission effectiveness [8].

### Lesson 2—Multidisciplinary team composition enhances operational flexibility

The inclusion of anaesthesia, emergency medicine, general practice and administrative competencies within a single ten-member Level 1 team provided flexibility to manage diverse needs, from complex acute presentations requiring specialist input to routine primary care and community assessment. Single-specialty or exclusively generalist EMT configurations risk capability gaps that cannot be remedied after deployment. Future team composition should be driven by rapid needs-assessment findings, with a core multidisciplinary structure augmented by mission-specific competencies [9].

### Lesson 3—Separating logistical from clinical functions protects medical capacity

The assignment of dedicated military units—structurally distinct from the medical component—for logistics, security and infrastructure allowed clinical staff to focus on patient care from mission arrival. In many humanitarian deployments, clinical personnel are diverted to infrastructure and security tasks during the first 72 h, precisely when the acute caseload is highest. The structural separation of logistical and clinical functions, formalised through the EMGA command structure, should be embedded in deployment standard operating procedures as a non-negotiable principle.

### Lesson 4—Epidemiological surveillance must begin on day one

The absence of standardised data-collection tools at mission outset represents the most correctable operational gap identified. A Level 1 EMT without prospective epidemiological capacity is limited both in its own adaptive management and in its contribution to national disease surveillance. Future military medical deployments should integrate World Health Organization-recommended surveillance instruments, including standardised consultation registers with disaggregated categories, from the first operational day [10].

### Recommendations

We propose three systemic recommendations for Senegalese military and national disaster planning. First, establish a Military Centre for Epidemiology and Public Health (Centre d’Épidémiologie et de Santé Publique des Armées, CESPA) responsible for pre-positioning essential supplies, maintaining updated risk cartography for flood-prone zones and coordinating surge deployment plans with civilian health authorities and regional partners [11]. Second, adopt the Multi-Cluster/Sector Initial Rapid Assessment (MIRA) as a mandatory pre-deployment planning instrument, enabling evidence-based resource allocation before a team is deployed [12]. Third, formalise aquatic-access protocols, including pirogue-based community reach, as a standard operational component for Level 1 EMTs in riverine flood environments rather than an improvised solution identified after arrival.

## An established military capacity in ongoing development

The Orkadiéré deployment did not represent an exceptional or improvised exercise of military capability. The Senegalese Armed Forces possess an established institutional capacity for disaster response, with prior deployments demonstrating the military’s role in national crisis management. The formal command architecture mobilised for this deployment—EMGA authority, DSSA coordination, zone-level operational management and formal reporting chains—reflects an existing doctrine rather than an ad hoc arrangement.

What the Orkadiéré experience reveals, however, is the gap between an existing capacity and a fully standardised one. The absence of prospective data-collection tools, the lack of pre-defined surveillance protocols and the informal nature of the lessons-derivation process point to areas in which the institutional framework can be strengthened. This documentation—the first published account of a Senegalese military EMT deployment in a flood context—contributes to that strengthening by making operational lessons available to planners within and beyond the Armed Forces.

Across the continent, the World Health Organization EMT initiative has progressively engaged African armed forces as contributors to national EMT rosters [5]. The Orkadiéré experience offers a concrete operational reference for this engagement in a Sahelian flood context, demonstrating both existing capacity and the standardisation gaps that the World Health Organization African Region initiative must address when supporting national EMT development in West Africa.

## Limitations

This commentary is based on operational field registers and two formal Comptes Rendus from a single 16-day deployment in one locality. The consultation figure (360) is a field-register tally reported through the military chain of command and was not independently verified; disaggregated clinical data were not systematically collected, limiting the ability to characterise the disease burden attributable to the disaster. The lessons presented reflect the deploying team’s operational judgment as expressed through the military after-action reporting process rather than an independent external evaluation. Geographic coverage was restricted to Orkadiéré and its immediate catchment, and the deployment period may not have captured delayed or secondary health consequences of flooding.

## Conclusion

The Orkadiéré deployment demonstrates that a well-composed and logistically supported military Level 1 EMT can deliver rapid, multidisciplinary and contextually adapted medical care in a West African flood emergency. The principal success factors were structured joint pre-deployment assessment, deliberate multidisciplinary team composition, structural separation of logistical and clinical functions and sustained civil-military coordination through an established military command framework. The principal correctable gap—the absence of integrated epidemiological surveillance from mission outset—is a priority for future deployment planning.

These lessons are offered as operational testimony rather than research findings. Their value lies in their specificity and proximity to practice. As climate change accelerates the frequency and severity of Sahelian floods, the capacity of African military health systems to contribute structured, accountable and scalable medical response will grow in strategic importance. Translating that capacity into standardised doctrine, validated surveillance tools and formalised inter-agency agreements is the necessary next step for the Senegalese Armed Forces and African military medicine more broadly.

## Funding

This work received no specific grant from any funding agency. The deployment described was conducted as part of the authors' routine duties within the Senegalese Armed Forces.

## Ethics

This commentary is based on operational field records. No patient identifiers were used. Institutional clearance was obtained from the Direction du Service de Santé des Armées.

## Artificial intelligence statement

The authors used Claude (Anthropic) to assist with manuscript restructuring, language editing and formatting for journal submission. The authors take full responsibility for the integrity of the work.

## Dissemination of results statement

This commentary has not been published previously in a peer-reviewed journal and is not under consideration elsewhere.

The operational observations described in the manuscript were reported internally through the Senegalese Armed Forces after-action reporting process in November 2024. These internal reports are not publicly available and do not constitute prior publication.

Following publication, the authors intend to disseminate the findings through:

• African emergency medicine and military medicine professional meetings;
• the Direction du Service de Santé des Armées, to inform EMT training and future deployment planning; and
• appropriate national and regional emergency-preparedness partners, including the WHO African Region EMT network.

## CReDiT author statement

AS Conceptualization, Methodology, Project administration, Supervision, Data curation and Investigation, Writing-original draft; ASN, MN, MLC Data curation and Investigation, Writing-review and editing. All authors approved the final manuscript and agree to be accountable for all aspects of the work.

## Declaration of competing interest

The authors declare that they have no competing financial, personal, or professional interests that could have influenced the work reported in this manuscript.

All authors have reviewed and approved this declaration.

## References

[1] Agence de Presse Sénégalaise (2024). https://aps.sn/.

[2] Organisation pour la Mise en Valeur du fleuve Sénégal (2024). Communiqué no. 9: crue du fleuve Sénégal 2024. https://www.omvs.org/.

[3] Baker M.S. (2007). Creating order from chaos: part II: tactical planning for mass casualty and disaster response. Mil Med.

[4] World Health Organization (2013).

[5] Balde T., Oyugi B., Mbasha J.-J., Kamara R., Martinez-Monterrey L.G. (2024). The emergency medical teams initiative in the WHO African region: a review of the development and progress over the past 7 years. Front Public Health.

[6] United Nations Office for the Coordination of Humanitarian Affairs (2008).

[7] Watson J.T., Gayer M., Connolly M.A. (2007). Epidemics after natural disasters. Emerg Infect Dis.

[8] Erlich T., Shina A., Segal D., Marom T., Dagan D. (2015). Preparation of medical personnel for an early response humanitarian mission—lessons learned from the Israeli defense forces field hospital in the Philippines. Disaster Mil Med.

[9] Beaussac M., Distinguin B., Turc J., Boutonnet M. (2020). Retour d’expérience des évacuations sanitaires MoRPHEE durant la COVID-19. Ann Fr Med Urgence.

[10] World Health Organization (2022).

[11] Dia I., Rambinintsaotra H.S. (2008).

[12] Inter-Agency Standing Committee (2015).

